# Sex Similarities in Postoperative Recovery and Health Care Contacts Within 14 Days With mHealth Follow-Up: Secondary Analysis of a Randomized Controlled Trial

**DOI:** 10.2196/periop.9874

**Published:** 2018-03-26

**Authors:** Maria Jaensson, Karuna Dahlberg, Ulrica Nilsson

**Affiliations:** ^1^ School of Health Sciences Faculty of Medicine and Health Örebro University Örebro Sweden

**Keywords:** sex, mHealth, telemedicine, mobile phone, cell phone, patient outcome assessment, postoperative complications, postoperative period

## Abstract

**Background:**

Previous studies have shown that women tend to have a poorer postanesthesia recovery than men. Our research group has developed a mobile phone app called Recovery Assessment by Phone Points (RAPP) that includes the Swedish Web version of the Quality of Recovery (SwQoR) questionnaire to monitor and assess postoperative recovery.

**Objective:**

The aim of this study was to investigate sex differences in postoperative recovery and the number of health care contacts within 14 postoperative days in a cohort of day-surgery patients using RAPP.

**Methods:**

This study was a secondary analysis from a single-blind randomized controlled trial. Therefore, we did not calculate an a priori sample size regarding sex differences. We conducted the study at 4 day-surgery settings in Sweden from October 2015 to July 2016. Included were 494 patients (220 male and 274 female participants) undergoing day surgery. The patients self-assessed their postoperative recovery for 14 postoperative days using the RAPP.

**Results:**

There were no significant sex differences in postoperative recovery or the number of health care contacts. Subgroup analysis showed that women younger than 45 years reported significantly higher global scores in the SwQoR questionnaire (hence a poorer recovery) on postoperative days 1 to 10 than did women who were 45 years of age or older (*P*=.001 to *P*=.008). Men younger than 45 years reported significantly higher global scores on postoperative days 2 to 6 than did men 45 years of age or older (*P*=.001 to *P*=.006). Sex differences in postoperative recovery were not significant between the age groups.

**Conclusions:**

This study found sex similarities in postoperative recovery and the number of health care contacts. However, subgroup analysis showed that age might be an independent factor for poorer recovery in both women and men. This knowledge can be used when informing patients what to expect after discharge.

**Trial Registration:**

ClinicalTrials.gov NCT02492191; https://clinicaltrials.gov/ct2/show/NCT02492191 (Archived by WebCite at http://www.webcitation.org/6y2UtMbvz)

## Introduction

Previous research has shown that women seem to have a poorer quality of postanesthesia recovery than men [1,2]. Even though women emerged faster from general anesthesia [1-3], women reported higher pain scores in the postanesthesia care unit (PACU) and in the first 3 days after surgery, and they also experienced more postoperative nausea and vomiting, as well as longer stays in the PACU, than did men [1]. Physical differences might explain these observed differences [1]. Also worth noting is that there may be gender role expectations resulting in men being less willing than women to report pain [4]. However, a weakness is that previous studies reporting patients’ recovery measured this only 2 to 3 times postoperatively [1,2,5-7]. As well, there is no consensus regarding on which day or days it is most important to follow up. Furthermore, patients experience several barriers to self-management during their recovery [8]. This may be one reason for unplanned health care contacts [9] and, according to one study, the most common reason was postoperative pain [10]. Another reason can be that follow-up after anesthesia and surgery is not performed routinely or as a telephone call on postoperative day 1 or 2 [11]. One way to follow up after surgery is to use mHealth solutions [12-14] to increase patients’ satisfaction [12,14] and to facilitate postoperative follow-up [12,15].

Evidence is lacking with respect to daily potential sex differences in postoperative recovery and the number of health care contacts. Therefore, the aim of this study was to investigate, through use of an mHealth solution, whether there were any sex differences in postoperative recovery within 14 postoperative days in a cohort of day-surgery patients in Sweden.

## Methods

### Study Design and Participants

This was a secondary analysis of data from a prospective, single-blind, multicenter, randomized controlled trial performed at 4 different day-surgery settings in Sweden. We carried out this study in accordance with the study protocol [16] and obtained approval from the regional ethical review board in Uppsala, Sweden (2015/262) [17]. The trial was registered with ClinicalTrials.gov (NCT02492191).

Participants received written information about the study before the planned surgery. Oral information was provided by the research nurse, who also was responsible for participant inclusion on the same day as surgery, and for collecting oral and written consent from all participants. Inclusion criteria were undergoing day surgery, being able to understand the spoken and written Swedish language, having access to a mobile phone, and being 18 years of age or older. Exclusion criteria were having memory impairment, visual impairment, or alcohol or drug abuse, or undergoing a surgical abortion.

The secondary aim of the randomized controlled trial was to investigate postoperative recovery. This paper presents only the participants who were randomly allocated to the intervention group [16]. In the intervention group, an app called Recovery Assessment by Phone Points (RAPP), which includes the Swedish Web version of the Quality of Recovery (SwQoR) questionnaire [13,14,18], was installed on the participant’s own mobile phone. No personal data were registered in the app. Every participant got a unique study code, and only the research team had access to study codes. The participant was instructed in how to report postoperative recovery daily for 14 days, starting from postoperative day 1. An additional function in the app was the possibility for the participant to be contacted by a nurse. Every day the app presented the question “Do you want to be contacted by a nurse? [Answer yes or no]”. If requested, a registered nurse, from the department where the surgery had been performed, called within 24 hours (on weekdays).

### Outcomes

The primary end point for this study was postoperative recovery assessed by the SwQoR questionnaire. Reliability and validity tests have provided sufficient evidence that the SwQoR questionnaire is appropriate to use for day-surgery patients [13,14,18,19] and is clinically feasible for systematic follow-up over time during postoperative recovery [19]. The SwQoR questionnaire comprises 24 items measuring postoperative recovery to be reported on an 11-point response scale, ranging from 0, “none of the time,” to 10, “all of the time” [19]. Guided by the main study, in this substudy, the SwQoR questionnaire had a possible global score ranging from 0, “excellent quality of postoperative recovery,” to 240, “extremely poor quality of recovery,” with cutoff values of less than 31 at day 7 and less than 21 at day 14 indicating good recovery [17].

On postoperative day 14, the participants answered a study-specific, paper-based questionnaire including yes/no questions (n=5) and the number of and reasons for all surgery-related health care contacts with primary care, an emergency department, Sweden’s 24-hour helpline (1177), an outpatient hospital, and contact via RAPP. We chose a 14-day follow-up because most care contacts are reported to be made in the first 2 weeks after day surgery [20].

We also recorded the following data: sex, American Society of Anesthesiologists (ASA) classification, type of surgery, type of anesthesia, duration of surgery, and time spent in the PACU.

### Statistical Analysis

We have presented the sample size calculation elsewhere; we did not calculate an a priori sample size regarding sex differences [16]. Descriptive statistics of baseline characteristics (age, sex, ASA classification, type of surgery, duration of stay at the PACU) were analyzed as the number, percentage, or mean (SD). We regarded missing answers in the returned questionnaires regarding health care contacts as no contact (scored 0). We tested continuous data for normality using the Shapiro-Wilk test. In this study, when analyzing the overall level of recovery, we used the global score. Guided by earlier studies [21-23], we used the mean (SD) of SwQoR scores. To compare differences between men and women, we used chi-squared, Student *t* test, and Mann-Whitney *U* test, as appropriate. We analyzed various subgroups to determine differences between types of surgery (general surgery, urology, and gynecology vs orthopedic and hand surgery) and age groups (<45 years vs ≥45 years, guided by the mean age in this study’s population). To determine differences between age groups, we used the mean value as the cutoff. To assess clinical significance, we analyzed the Cohen *d* effect size (small effect: 0.2-0.5; moderate effect: 0.5-0.8; large effect: >0.8) [24].

For statistical analyses, we used IBM SPSS Statistics version 24 for Windows (IBM Corporation). A *P* value <.01 was considered statistically significant in all analyses.

## Results

### Participants

We enrolled patients between October 2015 and July 2016 and assessed 1796 patients for eligibility. In all, we excluded 770 patients before randomization for various reasons, as described elsewhere [17]. We randomly assigned the remaining 1027 patients to either the RAPP intervention or the control group. The RAPP group consisted of 513 patients, of whom 19 did not receive the intervention, leaving a total of 494 patients (n=220, 44.5% men and n=274, 55.5% women). Of these, 127 men and 215 women returned the questionnaire regarding health care contacts.

There were no significant differences between men and women in terms of age, ASA classification, duration of surgery, or time spent in the day-surgery unit. There were significant differences in type of anesthesia, type of airway management, and type of surgery between men and women (*P*<.001; Table 1).

**Table 1:** Demographic data, patient characteristics, and anesthetic and surgical factors.

Characteristics		Men (n=220)	Women (n=274)	*P* value
**Age (years)**				
	Mean (SD)	44.13 (15.09)	45.49 (14.87)	.31^a^
	<45, n (%)	115 (49.8)	116 (50.2)	
	≥45, n (%)	104 (39.7)	158 (60.3)	
**American Society of Anesthesiologists classification, n (%)^b^**				.45^c^
	I	115 (47.5)	127 (52.5)	
	II	61 (41.5)	86 (58.5)	
	III	6 (54.5)	5 (45.5)	
**Type of anesthesia, n (%)**				.004^c^
	General anesthesia	176 (48.6)	186 (51.4)	
	Regional or local anesthesia	35 (32.7)	72 (67.3)	
**Type of airway management, n (%)**				.001^c^
	Endotracheal tube	42 (54.5)	35 (45.5)	
	Laryngeal mask	131 (49.1)	136 (50.9)	
	Mask	1 (16.7)	5 (83.3)	
	Spontaneous breathing	37 (31.1)	82 (68.9)	
**Type of surgery, n (%)^d^**				<.001^c^
	Orthopedic	75 (46.9)	85 (53.1)	
	General	60 (47.6)	66 (52.4)	
	Hand	50 (43.1)	66 (56.9)	
	Ear, nose, or throat	28 (53.8)	24 (46.2)	
	Gynecologic	N/A^e^	26 (100.0)	
	Eye	3 (60.0)	2 (40.0)	
	Urologic	3 (100.0)	N/A	
	Dental	N/A	2 (100.0)	
Duration of surgery (minutes), mean (SD)		43.61 (30.20)	37.92 (28.90)	.16^f^
Time spent in day-surgery unit before discharge (hours), mean (SD)		2.28 (1.66)	2.35 (1.82)	.67^a^

^a^Independent *t* test.

^b^Missing values for men: n=38; women: n=56.

^c^Chi-squared test.

^d^Missing values for men: n=1; women: n=3.

^e^N/A: not applicable.

^f^Mann-Whitney *U* test.

**Table 2:** Response rate for the Swedish Web version of the Quality of Recovery questionnaire.

Postoperative day	Response rate, n (%)	
	Men (n=220)	Women (n=274)
1	186 (84.5)	243 (88.7)
2	173 (78.6)	232 (84.7)
3	169 (76.8)	224 (81.7)
4	154 (70.0)	226 (82.4)
5	151 (68.6)	212(77.3)
6	149 (67.7)	207 (75.5)
7	140 (63.6)	201 (73.3)
8	137 (62.2)	199 (72.6)
9	136 (61.8)	189 (68.9)
10	128 (58.1)	182 (66.4)
11	127 (57.7)	178 (64.9)
12	117 (53.1)	187 (68.2)
13	129 (58.6)	191 (79.7)
14	117 (53.1)	167 (60.9)

### Comparisons by Sex and Age

There were no significant differences between men and women in their replies to items of the SwQoR questionnaire except on postoperative day 1, in which women scored higher than men in *dizziness* (*P*=.002, effect size 0.28), and on postoperative day 4, in which women scored higher on *more sleeping difficulties* (*P*=.003, effect size 0.30). On postoperative day 12, men scored higher than women on *reddened surgical wound* (*P*=.006, effect size 0.20).

The response rate decreased over time in both men and women (Table 2).

**Figure 1:**
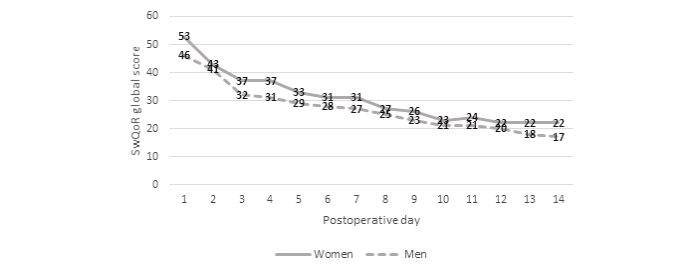
Global score (mean) for the Swedish Web version of the Quality of Recovery (SwQoR) questionnaire for men and women (higher scores indicate poorer recovery).

The SwQoR global score decreased over time. The mean score for men was 46 (SD 34) on postoperative day 1 and 17 (SD 21) on postoperative day 14. Corresponding numbers for women were 53 (SD 36) on postoperative day 1 and 22 (SD 28) on postoperative day 14. There were no significant differences in the global score between the sexes in postoperative recovery at any time point. Men had a global score below 30 at postoperative day 5, and women had a global score below 30 at postoperative day 8 (Figure 1).

When analyzing differences in items in the SwQoR questionnaire between the sexes in the 2 age groups, we found that women (<45 years) scored significantly higher (ie, poorer recovery) than men on the following items: *nausea or vomiting* on postoperative day 1 (*P*=.003, effect size 0.43) and postoperative days 3 and 4 (*P*=.001 and *P*=.003, effect size 0.43 and 0.53, respectively); *anxiety* on postoperative day 1 (*P*=.006, effect size 0.39); *dizziness* on postoperative day 1 (*P*=.002, effect size 0.43) and postoperative days 3 to 5 (*P* range .001 to .005, effect size range 0.39 to 0.54); *sleeping difficulties* on postoperative day 4 (*P*=.005, effect size 0.48) and postoperative day 8 (*P*=.008, effect size 0.36); and *headache* on postoperative day 9 (*P*=.002, effect size 0.61) and postoperative day 13 (*P*=.008, effect size 0.39). Women 45 years and older of age scored higher on the items *having difficulty returning to work or usual home activities* on postoperative day 1 (*P*=.01, effect size 0.38) and *having difficulty taking care of my personal hygiene* on postoperative day 2 (*P*=.01, effect size 0.30).

Men scored significantly higher (ie, poorer recovery) on the items *having trouble breathing* (*P*=.001, effect size 0.45), *sore throat* (*P*=.01, effect size 0.34), and *fever* (*P*=.007, effect size 0.24) on postoperative day 10. Also, men scored higher than women on *reddened surgical wound* (*P*=.01, effect size 0.24) on postoperative day 12 *.*

**Figure 2:**
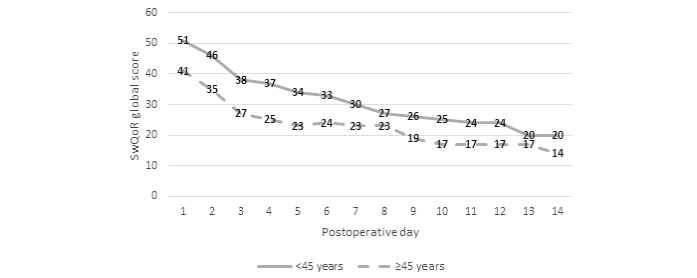
Differences in global score (mean) for the Swedish Web version of the Quality of Recovery (SwQoR) questionnaire by age for men (higher scores indicate poorer recovery). Differences between postoperative days 2 to 6 were statistically significant (*P* range .001 to .006).

**Figure 3:**
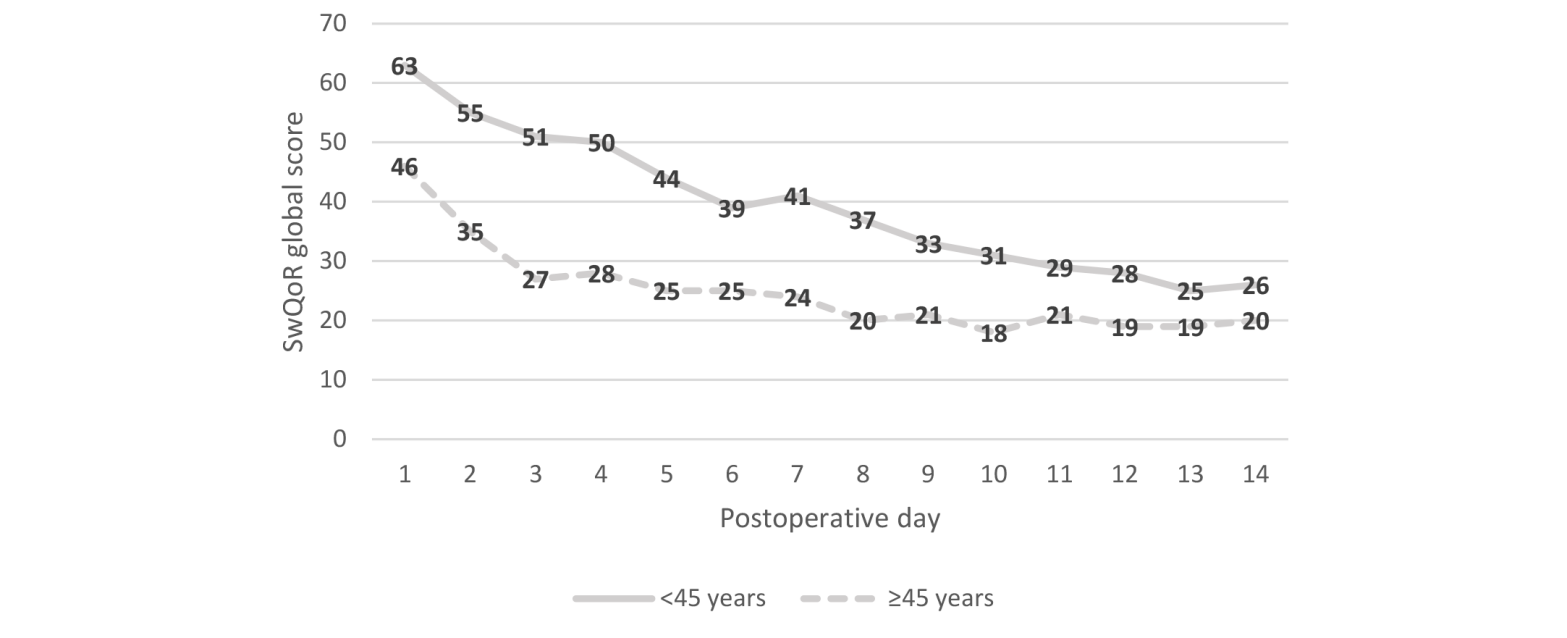
Differences in global score (mean) for the Swedish Web version of the Quality of Recovery (SwQoR) questionnaire by age for women (higher scores indicate poorer recovery). Differences between postoperative days 2 to 10 were statistically significant (*P* range <.001 to .008).

When analyzing the differences in SwQoR global score by age group (<45 years and ≥45 years), we found that men and women had somewhat similar recovery profiles. Younger men (<45 years) reported significantly higher global scores (ie, poorer recovery) on postoperative days 2 to 6 (*P* range .001 to .006) than did men 45 years of age or older (Figure 2). Women younger than 45 years reported significantly higher global scores (ie, poorer recovery) on postoperative days 1 to 10 than did women 45 years of age or older (*P* range <.001 to .008; Figure 3). A higher proportion of older women (≥45 years) than younger women (<45 years) had undergone orthopedic and hand surgery (n=98, 64.9% vs n=53, 35.1%) and general gynecologic surgery (n=48, 52.2% vs n=44, 47.8%). For men, the proportions for surgery were somewhat the same: a higher proportion of older men (≥45 years) than younger men had undergone general or urologic surgery (n=42, 66.7% vs n=22, 33.3%). The proportions for orthopedic and hand surgery were 42.7% (n=53) for older men versus 57.3% (n=71) for younger men. Finally, higher proportions of younger women (n=19, 67.9%) and younger men (n=22, 71.0%) had ear, nose, and throat surgery, eye surgery, or dental surgery than did the older age groups (n=9, 32.1% of older women and n=9, 29.0% of older men).

**Table 3:** Comparison of unplanned health care contacts (n=342).

Type of contact		Men (n=127)^a^	Women (n=215)^a^	*P* value
**Primary health care**				
	Number of persons, n (%)	4 (3.1)	12 (5.6)	.30^b^
	Number of contacts^c^	4	12	.30^d^
**Emergency department**				
	Number of persons, n (%)	4 (3.1)	8 (3.7)	.78^b^
	Number of contacts^c^	4	8	.78^d^
**Swedish 24-hour helpline (1177)**				
	Number of persons, n (%)	6 (4.7)	22 (10.2)	.07^b^
	Number of contacts^c^	7	26	.07^d^
**Outpatient hospital visits**				
	Number of persons, n (%)	7 (5.5)	16 (7.4)	.49^b^
	Number of contacts^c^	7	24	.46^d^
Phone call to the day-surgery department, n		1	1	
**Contact request via RAPP^e^app**				
	Number of persons, n (%)	17 (13.3)	37 (17.2)	.35^b^
	Number of contacts^c^	21	40	.36^d^
**Sum of unplanned contacts**				
	Number of persons, n (%)	25 (19.6)	67 (31.1)	.02^b^
	Number of contacts^c^	43	110	.03^d^

^a^Missing questionnaires for men: n=93; women: n=59.

^b^Chi-squared test.

^c^Unless otherwise specified, 1 contact per person was made.

^d^Mann-Whitney *U* test.

^e^RAPP: Recovery Assessment by Phone Points.

When comparing differences in SwQoR global scores by sex in the 2 age groups (<45 years and ≥45 years), we found no significant differences between the sexes at any time during postoperative days 1 to 14.

There were no statistical differences between sexes in health care contacts (planned or unplanned). Both men and women had most of their health care contacts via RAPP (21/43, 48.8% of all contacts for men and 40/110, 36.4% of all contacts for women; Table 3).

## Discussion

### Principal Findings

This study evaluated patients’ postoperative recovery during the first 14 postoperative days using RAPP, an mHealth solution. To our knowledge, this type of follow-up has never been performed previously. The focus of this study was sex differences, and the results showed no significant differences in postoperative recovery, either in the global score of SwQoR during the first 14 postoperative days or in health care contacts. In individual items, there were sex differences in only 3 of the 24 items, *dizziness* and *sleeping difficulties* on postoperative day 1 and *reddened surgical wound* on postoperative day 12.

The absence of differences between men and women in this study is in line with an Icelandic study using the 40-item Quality of Recovery (QoR-40) questionnaire, investigating 427 men and women undergoing day surgery [5]. Their results and ours are, however, in contrast with other studies showing sex differences, reporting women to be prone to poor postoperative recovery [1-3,25]. The underlying mechanism for the absence of sex differences in our study is not clear, and there may be several possible explanations. To mention a few, there could be cultural differences, or our findings may be a result of awareness of possible sex difference and implementation of evidence-based medical guidelines in clinical practice, such as preventing postoperative symptoms such as nausea and pain.

It may be that using mHealth is more beneficial for women. If so, this is consistent with a study investigating a telehealth intervention, showing that women in the intervention group had lower incidences of depression, fatigue, sleeping difficulties, and pain after coronary artery bypass surgery [26]. The possibility of reporting the postoperative recovery process on a daily basis has been shown to significantly decrease scores in SwQoR on individual items and to lower global scores compared with a control group [17]. On the last day of follow-up in our study, the response rate decreased; however, 60.9% of the women still reported their postoperative recovery. Another explanation may be that the intervention itself increased the feeling of self-efficacy; thus, it may have lessened any potential difference between men and women. Also, the patients could at any time press the button if they wished to be contacted by a nurse. This may have given a sense of security. However, Hyde [27] stated, in a review of gender differences and similarities, that gender differences in emotional experience are small, or in many cases, trivial, that there still exists a stereotype that portrays women as the emotional ones, and that there are large gender differences in emotions such as fear and anxiety. In our study, we also found similarities between the sexes in number of health care contacts, which is in line with an earlier study investigating predisposing factors for emergency department visits, which found no sex differences in such visits after surgery [28].

We assessed postoperative recovery in this study using the patients’ own mobile phones. The benefit of using e-assessment with a mobile phone is familiarity with the technology, which makes it easy to use [14]. Previous research has shown that barriers to using mobile technology can depend on one’s sex, among other factors, indicating that women have higher levels of anxiety and technophobia than men [29]. Therefore, the use of an app in relationship to the sexes and postoperative recovery needs to be investigated further.

Postoperative pain has been reported to be a common symptom during recovery at home [10]. SwQoR measures how often (from none of the time to all of the time) a symptom, feeling, or impaired ability occurs and not how severe a feeling or symptom is. It is not to be confused with a numeric rating scale measuring, for example postoperative pain. In this study both men and women patients reported pain from the surgical wound to be present most of the time, especially on the first postoperative days. One study investigating patients’ symptom management techniques after orthopedic day surgery reported that patients managed postoperative pain using different strategies, including taking pain medications, using ice to relieve pain and induce numbness, and reducing food and drink so they wouldn’t have to get up and move [8]. In respect of that result, it is likely that the sense of feeling relaxed and comfortable, as well as having a feeling of general well-being, and the difficulty in taking care of one’s personal hygiene and in returning to work or usual home activities may be interrelated with the patients’ postoperative pain in this study.

Our study showed that women 45 years and older reported significantly better postoperative recovery (hence, lower global scores on the SwQoR). The effect of the menstrual cycle phase on overall postoperative recovery have been investigated, showing that premenopausal women reported higher pain scores and had poorer recovery according to their scores [1]. This study also showed that men 45 years and older reported significantly lower global scores (ie, better recovery) on postoperative days 2 to 6. On the other hand, both younger women and younger men reported poorer recovery (ie, higher global scores on the SwQoR) in the first week after discharge. It may be argued that the cutoff used in this study was not appropriate. Different age cutoffs have been used when investigating younger and older patients—for example, less than 52 years [3] or less than 65 years [30]. This study’s result is somewhat in line with a large-scale study including 17,638 day-surgery patients, which found that elderly patients (ie, >65 years) had a lower incidence of any postoperative event (eg, pain, nausea and vomiting, shivering, and agitation) measured in the PACU and in the ambulatory surgical unit (adjusted odds ratio 0.43). However, the elderly patients had mostly undergone ophthalmologic surgery, which causes minimal postoperative pain [30]. The role of age, sex, and postoperative recovery needs further investigation. It is possible that this study’s results depended on the presence of generation gaps, attitudes, and gender role expectations. This Swedish sample may have had fewer gender role expectations.

A total of 158 (57.6%) of the women and 104 (47.2 %) of the men were 45 years of age or older. As a result of these differences, we analyzed the type of surgery, in case the younger population was confounded by the distribution of type of surgery. The analysis showed significant differences between the groups. Hence, there is a possibility that surgery and type of anesthesia can also be confounders related to the nonsignificant findings between the sexes.

### Study Limitations

The absence of significant differences between the sexes could have been due to the small sample size, and there might be a type II error. We calculated the sample size for the primary outcome, the cost effectiveness of RAPP [9,16]. However, the sample size in this study is almost the same as in other studies reporting sex differences [1,2]. Another limitation we acknowledge is that the patients did not report any baseline SwQoR scores. Patient-reported outcome after surgery and anesthesia is of great interest for health care professionals, as well as for the patient. The question is not why, but when and how patient-reported outcomes should be measured. There are also some concerns regarding how to compare results between different studies. Therefore, this study’s result must be interpreted with caution, and the results between studies are difficult to compare. Different instruments have been used in different studies, such as QoR-40 [5,6,22], the Postdischarge Surgical Recovery Scale [31,32], and the Postoperative Quality Recovery Scale [33]. These instruments were developed to be used with inpatients [33], outpatients [32], or both inpatients and outpatients [21]. In addition, the wording of items differs: usually there is a mix of positively and negatively worded items in an instrument [34]. In the SwQoR questionnaire, all items are negatively worded [18], and this construction is consistent with visual analog scales, which are anchored by two extreme values [35]. The SwQoR global score is also anchored by two values, 0 (excellent recovery) and 240 (poor recovery). The QoR-40 [21] and the Postoperative Quality Recovery Scale [33] were developed to be analyzed in dimensions. SwQoR evaluates the patient’s recovery on an item level, in the belief that the patient needs to be cared for according to which individual item indicating distress is disturbing. However, having said this, the possibility of analyzing global scores may offer an insight into the overall recovery process and be a surrogate measure for quality in the recovery process.

### Conclusions

This study indicated to that there are similarities in postoperative recovery and health care contacts between men and women. However, subgroup analysis showed that age may be an independent factor for poorer recovery in women and men. This knowledge can be used when informing female patients what to expect after discharge.

## Abbreviations

ASA: American Society of Anesthesiologists
PACU: postanesthesia care unit
QoR-40: 40-item Quality of Recovery
RAPP: Recovery Assessment by Phone Points
SwQoR: Swedish Web version of the Quality of Recovery
